# Health, ecology and the microbiome

**DOI:** 10.7554/eLife.47626

**Published:** 2019-04-17

**Authors:** S Andrew Inkpen

**Affiliations:** Department of PhilosophyBrandon UniversityBrandonCanada

**Keywords:** Philosophy of Biology, Philosophy of Science, Microbiome, Ecology, Health, Holobiont

## Abstract

Advances in microbiomics have changed the way in which many researchers think about health and disease. These changes have also raised a number of philosophical questions around these topics, such as the types of living systems to which these concepts can be applied. Here, I discuss the human microbiome from two perspectives: the first treats the microbiome as part of a larger system that includes the human; the second treats the microbiome as an independent ecosystem that provides services to humans. Drawing on the philosophy of medicine and ecology, I explore two questions: i) how can we make sense of disease and dysfunction in these two perspectives? ii) are these two perspectives complimentary or do they compete with each other?

Back in 2007, in a playful special feature of the journal *Environmental Microbiology* titled 'Crystal Ball', three leading microbiome researchers foresaw a time when medical insurance cards would have two chips: one containing information about our genome, the other information about our microbiome (the communities of microbial species we harbor on and in our bodies). They predicted that changes to our microbiomic profile would indicate a predisposition to diseases such as obesity (Ley et al., 2007).

Although yet to fully materialize, their speculations were prescient in envisioning a time when microbiomics − that is, the study of the genomes of microbiomes − would be central to the study of human health and disease. Moreover, advances in this field have challenged many philosophical assumptions about health, and the related concepts of individuality, function and evolution. In this article, I discuss the philosophical issues that arise in relation to the idea of the healthy human microbiome.

## Ecological systems and health

A basic question is: what kinds of living systems have the capacity for being healthy? Traditionally, concepts of health and disease apply to biological individuals. Cells, plants and animals (including humans) can be healthy or diseased, but what about ecological systems such as populations, communities and ecosystems (Lackey, 2001)? In the 1990s, this question dominated research in ecology, environmental science and sustainability, with ‘ecosystem health’ being an important goal for those involved in environmental management. Proponents argued that if we intervene in ecological systems, we should aim to improve their health, rather than restore their natural state (Costanza et al., 1992). After 10 years of debate, however, a consensus emerged that only individuals can be healthy and diseased, and since ecosystems are not individuals, those concepts do not properly apply to them (Jamieson, 1995).

What makes us think that only individuals have the capacity for health?

Two decades of research in microbiomics have raised anew the question of whether ecological systems can be healthy and challenge the arguments in the previous paragraph. The correct theory of individuality is itself a contentious matter, and the idea that ecosystems are not individuals is debatable, and likely false (Bouchard and Huneman, 2013). But more fundamentally, what makes us think that only individuals have the capacity for health? Indeed, researchers frequently speak of the ‘healthy microbiome’, which implies that the concept of health applies to an ecological community. Would it not be sensible, then, to first evaluate the characteristics that classify a living system as possessing the capacity for health, and then decide which ecological systems meet these criteria? Is it even useful to speak of the healthy human microbiome? The answer to these questions depends on the three different perspectives on this matter that exist within the microbiomics community.

First, the ‘holobiont perspective’ (a holobiont being a cluster of different species that form ecological units) treats the microbiome and its human host as a single individual, with the microbiome often being viewed as an organ or organ system − that is, the microbiome is a functional part of this individual (Hutter et al., 2015; Clarke et al., 2014). Second, the ‘ecological perspective’ treats the microbiome and host as a single ecosystem. Highlighting the connection between human medicine and restoration ecology, one team of researchers wrote: "[the] emerging view of humans as ecosystems raises the question of whether approaches developed to improve the health of natural ecosystems may help to advance gut medicine” (Orr et al., 2018). Third, the ‘ecosystem services perspective’ treats the microbiome as an ecosystem that provides ‘services’ to the human. Like any ecosystem, the host has some control over the structure of this system and the functional benefits it provides (Foster et al., 2017).

## Three different perspectives

According to the holobiont and ecological perspectives, the microbiome is considered part of a larger system (the holobiont or the ecosystem) to which health is attributed (see Skillings, 2016) for a philosophical discussion). These perspectives are bolstered by a central finding: the communities of microbial species that live symbiotically with a human host are highly integrated into basic physiological processes, such as digestion and the development of the immune system (Human Microbiome Project Consortium, 2012; Inkpen et al., 2017).

This high degree of integration suggests that the holobiont or ecosystem itself could possess the capacity for health. Therefore, the microbial community in our gut is not just a part of our environment, as previously thought, but an essential part of our health. Philosophers of medicine have argued that we should thus break from tradition and extend our concept of health to cover these ‘dynamic functional units’ (Morar and Skorburg, 2018).

Nevertheless, being functionally integrated is not sufficient for a living entity to possess the capacity for health. When a philosopher of medicine says that an entity is healthy or diseased, they are making a claim about the internal functioning of the entity − things are either working as they should or they are not. This reasoning can also be applied to an ecological system. However, being in a diseased state does not imply that the entity is worse off, in the sense of having decreased wellbeing. For example, according to recent test results, something is wrong with my dog’s gall bladder, so she is not in perfect health. However, this is not severe enough for her to have perceivable symptoms: in other words, she is unhealthy, but she is not worse off. Likewise, a tree may have Dutch elm disease (a fungal disease transmitted by beetles) and yet, not experience decreased wellbeing due to a lack of the cognitive capacities required. So, this minimal sense of disease, which is important in biology and for theories of pathology, should not be conflated with substantive judgments about illness in humans.

To determine if a microbiome is healthful or unhealthful, we need to analyze the health of its host.

According to this minimal sense, health is not simply a descriptive claim about functional integration. We have to distinguish why some ways of being functionally integrated are healthy while others are not. Philosophers explain this in terms of functional normativity: when something has gone wrong, we say that it is dysfunctional. If I have heart disease, then my heart is dysfunctional. But how do we make sense of dysfunction? And what systems possess the capacity for this ascription? This is a controversial matter.

The most natural way to explain dysfunction in living systems in this sense is to appeal to natural selection: a part fails to perform its function if it fails to do what it was selected to do (Griffiths and Matthewson, 2016): my broken hand is dysfunctional because it does not do what it was selected to do − grasp things. But that does not explain dysfunction in ecosystems, since most of them are not subject to natural selection and thus their ‘parts’ cannot have dysfunctions as such (Doolittle and Inkpen, 2018). Hence, despite being highly functionally integrated, most ecological systems and holobionts cannot be health subjects.

A different way to explain dysfunction would be through functional efficiency: a part is not working as well as it should when its functional efficiency (its contribution to a specific system, such as metabolism) drops below a certain level (Hausman, 2014). We justify our choice of level by appealing to the functional efficiency of the same parts of other systems in a wider population. This second account of dysfunction is lenient. Assuming that holobionts or ecosystems make up populations of similarly functionally-organized systems, we should allow that concepts of health and disease apply to these living systems. Natural selection is not required.

The ecosystem services perspective treats the microbiome as an independent ecosystem that provides its host with services required for life and for individual wellbeing (Calow, 1995; Díaz et al., 2018). In the case of human hosts, the services supplied by the microbiome include help with digestion, the development of the immune system and protection against pathogens. Strictly speaking, philosophers of medicine use the word healthy to describe a state of internal functioning of an entity. With the ecosystem services perspective to the microbiome, on the other hand, we are interested in external factors, and a microbiome that is functioning properly (that is, a microbiome that is supplying services to its host) is said to be ‘healthful’ rather than healthy (Boorse, 2014). In this view, we draw a distinction between something that has gone wrong within an individual and something that has gone wrong with its environment. For example, very high temperatures are unhealthful for humans, but they do not constitute ill-health (although they may lead to it). To determine if a microbiome is healthful or unhealthful, we need to analyze the health of its host. While this approach may be new or unusual in microbiomics and many areas of biology, it is commonplace in traditional ecology, where it is used to how we calculate the services provided by any ecosystem that we benefit from (Díaz et al., 2018).

## Conflict or compromise?

All three perspectives share a desire for a richer understanding of human health in the ‘war no more’ approach (Lederberg, 2000). Rather than treating human health as a war against invading pathogens, it involves seeing a person as an ecological system, and the maintenance of health as a matter of management, restoration and sustainability.

So, should we see the three perspectives discussed in the previous section as competing or complementary? There may be some conflict between the holobiont perspective (in which the microbiome is essentially a human organ) and the ecosystem services perspective that could affect the classification of diseases: does a dysfunctional microbiome constitute a disease (like heart disease) or does it constitute an unhealthful environmental factor (like a polluted environment)? The ecological perspective and the ecosystem services perspective, on the other hand, are complimentary on the topic on microbiome health (and both have coexisted within traditional ecology for the last thirty years).

Microbiomics challenges us to think more expansively about health and what it means to be healthy. Philosophy can contribute to discussions about these questions, particularly questions about functional normativity and dysfunction. As previous debates about ecosystem health have demonstrated, asking such questions can be a productive two-way street.

## Note

This Feature Article is part of the Philosophy of Biology collection.
